# Curcumin primed ADMSCs derived small extracellular vesicle exert enhanced protective effects on osteoarthritis by inhibiting oxidative stress and chondrocyte apoptosis

**DOI:** 10.1186/s12951-022-01339-3

**Published:** 2022-03-09

**Authors:** Chen Xu, Zanjing Zhai, Hua Ying, Lin Lu, Jun Zhang, Yiming Zeng

**Affiliations:** 1 Shanghai Key Laboratory of Orthopedic Implants, Department of Orthopedics, Ninth People’s Hospital, Shanghai Jiao Tong University School of Medicine, Shanghai, 200011 China; 2 Department of Plastic and Reconstructive Surgery, Shanghai Ninth People’s Hospital, Shanghai Jiao Tong University School of Medicine, Shanghai, 200011 China

## Abstract

Osteoarthritis (OA) is a common joint disease caused by progressive articular cartilage degeneration and destruction. Currently, there are no disease-modifying agents officially approved for OA patients. In this study, curcumin was loaded into adipose tissue-derived mesenchymal stem cells (ADMSCs)-derived small extracellular vesicle (ADMSCs-sEV) to synergistically exert chondro-protective effects in vitro and in vivo. We found curcumin primed ADMSCs derived sEV (sEV-CUR) exhibited an enhanced protective effect compared with free curcumin and ADMSCs-sEV in TBHP-induced chondrocytes. Moreover, our study demonstrated sEV-CUR more effectively down-regulated TBHP-induced oxidative stress and chondrocyte apoptosis in vitro. In OA mice model, our results indicated that sEV-CUR showed an improved cartilage protection, as biweekly intra-articular injection of sEV-CUR more efficaciously alleviated oxidative stress and chondrocyte apoptosis in OA cartilage. Overall, our findings showed sEV-CUR exhibited enhanced chondro-protective effects and holds great potential on the recovery of articular cartilage loss and destruction in OA patients.

**Graphical Abstract:**

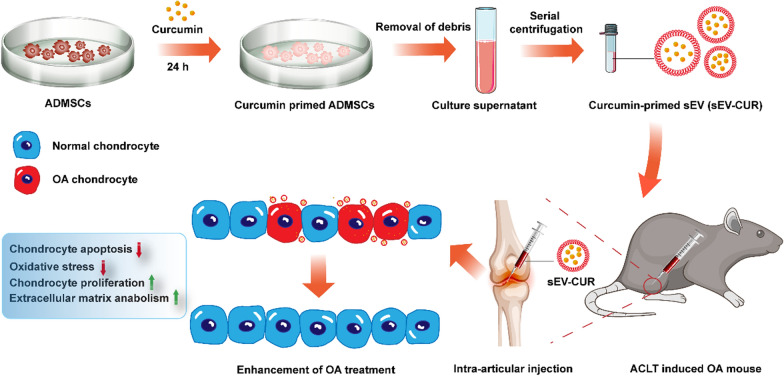

**Supplementary Information:**

The online version contains supplementary material available at 10.1186/s12951-022-01339-3.

## Introduction

Osteoarthritis (OA) is a common degenerative joint disease that manifests as degeneration and damage of articular cartilage, leading to pain and disability [1]. To date, OA affects more than 300 million globally and it is the primary reason for disability in the elderly. OA not only causes physical and psychological agony, but also results in a substantive socioeconomic burden [2]. Currently, there are no officially approved disease-modifying treatments [3].

The impaired homeostasis of chondrocyte plays an essential role in OA progression by secreting elevated quantities of proteolytic enzymes, such as aggrecanase (ADAMTS5) and matrix metalloproteinase 13 (MMP13), causing degeneration and damage of the articular cartilage [4]. Oxidative stress activated by different stress factors result in the release of inflammatory mediators (IL-1β, IL6, and TNFα), which lead to the imbalance of anabolism and catabolism, and ultimately bring about excessive chondrocyte apoptosis and articular cartilage destruction [5]. Recent studies demonstrated that oxidative stress and downstream signaling pathways are pivotal in the initiation and progression of age-related cartilage changes in OA [5, 6]. Moreover, inhibition of oxidative stress alleviated chondrocyte apoptosis and cartilage degeneration in various OA models [7–10]. Therefore, effective manipulation of oxidative stress and related chondrocyte apoptosis is essential to OA treatment.

Small extracellular vesicles (sEV) are natural lipid bilayer nanoparticles (30–150 nm) containing nucleic acid, proteins, lipids, and other biomolecules that take part in intracellular communication and regulating recipient cells behavior via the delivery of functional molecules [11, 12]. To date, the role and therapeutic potential of stem cell-derived sEV in cartilage repair and regeneration have attracted considerable attention worldwide [13]. Recently, some studies revealed that sEV derived from adipose tissue-derived mesenchymal stem cells (ADMSCs) promoted repair and regeneration of damaged tissues, alleviated degradation of aged organs, and maintained the homeostasis of the body [14–17]. In OA treatment, several studies indicated that MSCs-derived sEV (MSCs-sEV) significantly promoted chondrocyte proliferation in vitro and in vivo [18–21]. Wu et al. suggested that sEV derived from infrapatellar fat pad MSCs protected articular cartilage and ameliorated gait abnormalities in OA animal model [22]. In addition, Miguel et al. demonstrated ADMSCs derived sEV alleviated OA progression by downregulating osteoarthritic osteoblast senescence phenotype [23]. A recent study also revealed that ADMSC is a superior reservoir in sEV-based biomaterials for cartilage repair and regeneration, compared with bone marrow derived MSC (BMMSC) and synovium derived MSC (SMMSC) [24]. Therefore, ADMSCs were chosen as the source of sEV in our study. Intra-articular injection provides an appropriate administration method for MSC-sEV deployment. In previous studies, intra-articular injection of MSCs-sEV was performed at least once a week [18, 19, 25]. However, long-term and multiple intra-articular injection of sEV may result in the soft tissue injury and joint infection, limit their clinical application. Therefore, therapeutic drugs should be further loaded in MSCs-sEV to synergistically exert chondro-protective effects in OA cartilage. As we known, MSCs-sEV is an ideal nanoplatform for drug delivery [26–28]. First of all, MSCs-sEV can alleviate chondrocyte apoptosis and prevent OA progression by different mechanisms as described before [20–22]. Moreover, the lipid bilayer of sEV protects the lumen cargo and the nano-sized structure of sEV allows to cross different biological barriers. sEV have minimal tumorigenic risk and good biocompatibility. Given the above, ADMSCs-derived sEV (ADMSCs-sEV) can be used as an ideal drug delivery platform.

Curcumin is an excellent anti-oxidant agent which showed great therapeutic effects in the treatment of cartilage repair and regeneration [29, 30]. However, curcumin showed low water solubility and the fast clearance in the joint cavity, which limited the application and accurate control of curcumin dose in OA patient. To address this problem, curcumin is co-incubated with sEV, and curcumin primed sEV are used in damaged tissues repair and various degenerative diseases treatment for better bioavailability. Wang et al. indicated that curcumin primed sEV potently ameliorated neurodegeneration and cognitive function in Alzheimer’s disease mice by inhibiting Tau phosphorylation through AKT/GSK-3β signaling pathway [31]. Moreover, curcumin-loaded embryonic stem cell-derived sEV restored neurovascular unit following ischemia-reperfusion injury in mice [32]. Furthermore, curcumin primed sEV reversed LPS-induced inflammatory phenotypes and mitigated granulosa cell dysfunction [33]. In OA treatment, a recent study showed that curcumin treated MSCs-sEV attenuated cartilage destruction via regulating the miR-124/NF-κB and miR-143/ROCK1/TLR9 pathways in OA mice [34]. This work indicated the possible protective effect of curcumin primed MSCs-sEV in OA cartilage. However, a more comprehensive and meticulous experimental analysis need to be performed in future studies. In our study, we found curcumin primed ADMSCs derived sEV (sEV-CUR) more effectively promoted chondrocyte proliferation and ameliorated cell apoptosis in vitro, compared with free curcumin and ADMSCs-sEV. In addition, our results demonstrated that sEV-CUR exhibited an improved cartilage protective function, as biweekly intra-articular injection of sEV-CUR more efficaciously alleviated oxidative stress and chondrocyte apoptosis in OA cartilage. Finally, we found sEV-CUR effectively alleviated OA-related pain in mice 4 weeks after ACLT surgery. In conclusion, our results demonstrated that curcumin primed ADMSCs-sEV enhanced the therapeutic effect of curcumin and ADMSCs-sEV in OA cartilage with reduced injection frequency, and may be a potential therapy for OA patients in the future.

## Materials and methods

### Materials and reagents

Curcumin, bovine serum albumin (BSA), TBHP, and DMSO were obtained from sigma Sigma-Aldrich (St. Louis, MO, USA). ADMSCs were cultured in the serum-free ncMission hMSC Medium (RP02010, Nuwacell Biotechnologies Co., Ltd, China). Fetal bovine serum (FBS), collagenase II, 0.25% trypsin, 100 U/mL penicillin G and 0.1 mg/mL streptomycin were purchased from Gibco (NY, USA). Chondrocyte growth medium Dulbecco’s Modified Eagle Medium F-12 (DMEM/F12) and phosphate buffered saline (PBS) were obtained from Hyclone (Logan, UT, USA). sEV lipid fluorescent dyes DiI was purchased from Thermo Fisher Scientific (MA, USA). Annexin-V-FITC Apoptosis Detection Kit, 4′,6-diamidino-2-phenylindole (DAPI), EdU Cell Proliferation Kit with Alexa Fluor 488, and One Step TdT-mediated dUTP Nick-End Labeling (TUNEL) Apoptosis Kit were purchased from Beyotime Biotechnology (Jiangsu, China). The following monoclone antibodies and secondary antibody were obtained from Abcam (Cambridge, UK): anti-CD63, anti-tsg101, anti-GM130, anti-8-OHdG, anti-collagen II and anti-aggrecan. The primary antibody of cleaved caspase3 was obtained from Cell Signaling Technology (Danvers, MA, USA).

### Curcumin primed ADMSCs-derived sEV collection

First, sEV secreted from ADMSCs were used in our study. ADMSCs were isolated from C57 mice as described previously [24]. Briefly, for mice ADMSCs isolation, the inguinal adipose tissue was finely minced and digested in minimum essential medium-alpha (MEMα; Gibco) containing 0.1% collagenase I (Invitrogen) at 37 °C for 2 h. Then the lipid layers were removed after centrifugation, and the ADMSCs were resuspended and cultured in the serum-free ncMission hMSC Medium. The medium was replaced every 2 days. Then, ADMSCs were treated with or without curcumin (10 µM/L) for 24 h and sEV are isolated and purified from the supernatant by differential ultracentrifugation. Briefly, the culture medium was centrifuged at 300×*g* for 15 min and 2000×*g* for 30 min. Then, large MVs were removed by high-speed centrifugation (10,000×*g*) for 1 h and filtration through a 0.22 μm filter (Merck Millipore, MA, USA). Next, the supernatant was centrifuged at 100,000×*g* for 2 h (ultracentrifuge Optima XPN with a SW32 Ti rotor, Beckmann Coulter, USA). After removing the supernatant, the sEV pellet is resuspended in PBS, followed by another ultracentrifugation at 100,000×*g* for 2 h. All procedures were performed under 4 °C. Finally, the pelleted sEV and curcumin primed sEV (sEV-CUR) were resuspended in PBS.

### Characterization of curcumin primed sEV

To verify whether primed curcumin could alter the integrity of sEV, a series of experiments were performed. First, TEM was used for morphology observation of sEV and sEV-CUR. For TEM observation, sEV or sEV-CUR suspension was dropped onto a formvar-carbon-coated grid, and dried in air for 20 min. Next, the grids were rinsed with sterile PBS and fixed in 1% (w/v) glutaraldehyde for 5 min. Then the grids were rinsed with deionized (DI) water, and stained with 2% (w/v) uranyl oxalate for another 5 min. After drying, the microstructure of sEV or sEV-CUR were observed by TEM (Hitachi H-7650, Tokyo, Japan). Next, a nano flow cytometry (NFM, N30 NanoFCM, Xiamen, China) was used to detect the particle diameter distribution of sEV and sEV-CUR. To examine the expressions of surface markers in sEV and sEV-CUR, the protein samples were collected from sEV, sEV-CUR and ADMSCs using RIPA lysis buffer (Beyotime, Jiangsu, China). Briefly, after being incubated with 5% BSA for 1 h, the polyvinylidene difluoride (PVDF) membranes were incubated with primary antibodies including CD63 (1:1000), TSG101 (1:1000), and GM130 (1:1000) overnight at 4 ℃. Then, the membranes were incubated with HRP-labeled secondary antibody for 1 h and chemiluminescent signals were visualized by the Bio-RAD imaging system (Bio-RAD, CA, USA). The surface charge of sEV and sEV-CUR were detected by the nanoparticle analyzer (DelsaMax Pro, Beckman Coulter, USA).

To detect the release of free curcumin (CUR) and sEV-CUR in vitro, CUR and sEV-CUR containing 3.68 µg curcumin were solubilized in 1mL PBS (pH = 7.4, 37 °C) containing 0.5% carboxymethyl cellulose, and then added into the dialysis bag with a molecular weight cutoff of 2000 molecular weight. Next, the dialysis bag was immersed in a flask containing 200 mL of release medium and 0.5% (w/v) anionic surfactant sodium dodecyl sulfate. Samples were taken at different time points from inside the flask and the amount of released curcumin was detected and analyzed the fluorescence intensity of curcumin (Excited: 425 nm; Emitted: 530 nm) by a multifunctional microplate reader named LUX (Thermo Fisher Scientific, USA).

### Chondrocyte culture and treatments

Chondrocytes were isolated from healthy femoral head articular cartilage of 4-week-old C57 mice obtained from Sino-British Sippr/BK Lab Animal Ltd. (Shanghai, China). The animal use and care protocols were ethically approved by the Animal Use and Care Committee of Shanghai Jiaotong University. Mouse hip articular cartilage was digested with 0.25% trypsin for 30 min, and plated in Petri dishes containing 0.2% collagenase II at 37 °C for 4 h. Cell suspensions were centrifuged at 1200 rpm for 5 min and the cell pellets were resuspended in complete DMEM/F12 culture medium with 10% FBS. Primary chondrocytes were passaged at 80% confluence and the chondrocytes of second passage were used for the following experiments. To induced oxidative stress in chondrocytes, 20 µM/L TBHP was used to treat chondrocytes for 24 h. Then, TBHP-induced chondrocytes were treated with free curcumin (10 µM/L), sEV (1 × 10^9^ p/mL), and sEV-CUR (1 × 10^9^ p/mL) for 2 days to inhibit oxidative stress and related apoptosis.

### Cellular uptake assay

For cellular uptake assay, DiI was used to label sEV. First, sEV or sEV-CUR were incubated with 10 µM DiI for 30 min at 37 °C in dark. Next, the suspension was filtered by a 0.22 μm membrane and centrifuged at 100,000×*g* for 2 h to remove the uncombined dye. Then, the purified DiO-labeled sEV or sEV-CUR were incubated with mouse chondrocytes for 24 h under 5% CO_2_ at 37 °C. Subsequently, the chondrocytes were fixed with 4% PFA for 30 min and stained with DAPI for 5 min. The images in each time point (0 h, 30 min, 1 h, 2 h, 3 h, 6 h, 12 h, 24 h) were observed and captured by using a confocal microscopy (Leica Microsystems, Germany). Data and image analysis in each group was conducted using the IDEAS software (Luminex) as described previously [35, 36]. The data of mean fluorescence intensity in each time point was analyzed and compared by Graphpad Prism 8 software (CA, USA).

### Chondrocyte proliferation assay

The pro-proliferative ability of curcumin, sEV, and sEV-CUR on chondrocytes were examined by CCK8 assay and EdU staining assay. For CCK8 assay, 5 × 10^3^ mouse chondrocytes were seeded each well in 96-well-plate and then treated with TBHP and curcumin, sEV, or sEV-CUR for 2 days. Cytotoxicity was detected after 48 h. The cells were washed twice with PBS, and then 10 µL CCK8 solution was added to each well of the plate and incubated in the dark for 2 h at 37 °C. The absorbance of each well was measured at 450 nm using a microplate reader. All experiments were repeated thrice in sextuple. For EdU staining assay, 10 µM/L EdU solution was added to the DMEM/F12 culture medium. Then, the chondrocytes were washed with PBS thrice and fixed with 4% PFA for 30 min. Finally, chondrocytes in each group were captured by using a confocal microscopy.

### PCR analysis

After treated with free curcumin, sEV, or sEV-CUR in TBHP induced chondrocytes for 2 days, total RNA in each group was extracted by using the RNA Extraction Kit (Qiagen, Germany). After synthesis using the PrimeScriptTM RT Master Mix (Takara, Japan) from total RNA, the cDNA was amplificated by the SYBR Premix Ex TaqTM (Takara, Japan). Then, the RNA and cDNA products could be stored at − 80 °C for later investigation. Each experiment was performed in triplicate and experiments repeated thrice independently. A dissociation curve analysis was conducted for each quantitative PCR. Expression levels of the target genes were detected and analyzed by using a relative quantification approach (2^−ΔΔCt^ method) against β-actin levels.

### Immunofluorescence staining

After different treatments, chondrocytes in each group were fixed with 4% PFA for 30 min and permeablized with 0.025% Triton-X 100 for 15 min at room temperature. Then the chondrocytes were blocked with 5% BSA and incubated with primary antibodies overnight at 4 ℃. Subsequently, the chondrocytes were incubated with secondary antibodies for 1 h followed by 5 min incubation with DAPI for nuclear staining. Images in all groups were detected and captured by using a microscopy.

### Measurement of intracellular ROS

The level of ROS in each group of chondrocytes was quantified by the Reactive Oxygen Species Assay Kit. Briefly, 2′,7′-dichlorodihydrofluorescein diacetate (DCFH‐DA) is oxidized by ROS in viable chondrocytes to 2′,7′‐dichlorofluorescein (DCF) which is highly fluorescent at 530 nm. The chondrocytes were washed thrice with sterile PBS solution. Then, 10 µM/L DCFH‐DA was added to chondrocytes, and incubated for 30 min at 37 °C in the dark. After being washed thrice with sterile PBS solution, the relative level of fluorescence in each group was quantified by a microplate reader.

### Lipid peroxidation assay

After treated with free curcumin, sEV, or sEV-CUR in TBHP-induced chondrocytes, cells in each group were washed three times with sterile PBS solution, and total proteins were extracted with 100 µL RIPA. The lysates in each group were processed under ultrasonic and centrifuged at 12,000×*g* for 5 min. Then, the supernatants were quantified with BCA Protein Assay Kit. MDA equivalents were measured by thiobarbituric acid test according to the manufacturer’s instruction.

### Apoptosis analysis

For Annexin V-FITV apoptosis assay, after different treatments, the chondrocytes were washed with PBS thrice and trypsinized without EDTA. Removing the supernatant after centrifugation for 3 min at 1500 r/min, the chondrocytes were resuspended in 500 µL of binding buffer, and FITC-labeled Annexin V (20 µg/mL) and PI (50 µg/mL) (BD PharMingen, USA) were added, 5 µL each. The reaction proceeded in the dark for 15 min and the rate of apoptosis (%) was detected by flow cytometry (BD FACS ARIA II, SORP, USA). AnnexinV^+^PI^−^ and AnnexinV^+^PI^+^ cells were described as apoptotic chondrocytes [37]. For TUNEL staining, the chondrocytes in each group were incubated with TUNEL solution for 1 h by using a TUNEL staining kit at 4 ℃ overnight, and then incubated with DAPI for nuclear staining. Fluorescence Images in all groups were detected and captured by a fluorescence microscopy.

### Surgical induced mice OA model

To establish an in vivo OA model, 30 male C57 mice (N = 6 mice/group) underwent sham surgery or anterior cruciate ligament transection (ACLT) surgery were randomly and averagely divided into 5 groups: (1) Sham group; (2) ACLT + PBS group; (3) ACLT + CUR group; (4) ACLT + sEV group; (5) ACLT + sEV-CUR group. For the ACLT surgery, all mice were anaesthetised with isoflurane and the right knee joints of mice were shaved for aseptic operation. After opening the articular capsule, the anterior cruciate ligament was transected by a micro-scissor. For the sham surgery, the mouse joint was exposed by a medial capsular incision without any other operation, and then the surgical incision was closed. Frequent and long-term intra-articular injections could bring about many problems, such as soft tissues injuries and joint infection. Thus, reducing the injection frequency is essential for the clinical application of MSCs-sEV for OA patients. Thus, biweekly injection of PBS, sEV, or sEV-CUR was conducted in our study. 10 µL PBS or curcumin, sEV (1 × 10^9^ p/mL), or sEV-CUR (1 × 10^9^ p/mL) was intra-articularly injected into the joint cavity of the mice right knee biweekly. 4 weeks after treatments, all the mice were sacrificed and the right knee joints were harvested for following analysis.

### Histological evaluation and immunohistochemistry

Four weeks after treatments, Hematoxylin–Eosin (H&E) staining, Safranin O-Fast Green (S–O) staining, and Toluidine blue staining were performed to evaluate the cartilage degeneration and in each group. Following 4 weeks treatments, mice were killed by overdose pentobarbital, and the right knee joints were collected. The joints were fixed in 4% PFA for 24 h and then decalcified in 10% EDTA for 7 days. The tissues were dehydrated, embedded in paraffin, and cut to obtain serial 5-µm thick sections. Next, sections were stained with H&E, S–O or Toluidine blue staining dye. Finally, images in each group were captured by using a microscopy. Moreover, the level of cartilage degeneration was also assessed and quantified by the Mankin scoring system [38]. For immunohistochemistry (IHC) staining, the sections in each group were blocked with 3% hydrogen peroxide for 10 min and 5% BSA for 30 min. Next, the tissue sections were incubated with monoclonal antibodies against aggrecan (1:100), collagen II (1:100), 8‐OHdG (1:100), and cleaved caspase3 (1:100) overnight at 4 °C and HRP‐conjugated secondary antibody for 1 h, and visualization with 3,3‐diaminobenzidine chromagen or 3-amino-9-ethylcarbazole (Solarbio, China). Images in each group were captured using a microscopy.

### Pain measurements

Hind–paw withdrawal thresholds were detected to measure OA-related pain in our study [20, 39]. First, the mice were placed in an elevated metal grid cage with sufficient space to move their paws, while the rest body was restricted. After the mouse is acclimated to the apparatus, the right hind-paw withdrawal threshold of each mouse was analyzed by using an electronic von Frey instrument (model BIO-EVF4; Bioseb, Vitrolles France). Specifically, the probe tip of the instrument was gently placed perpendicularly into the mid-plantar surface of the paw, and steadily increasing pressure was applied until the hind paw was first lifted. This force is independent of the movements of the limb and the hind-paw withdrawal threshold was recorded as the required pressure to first lift the paw. The final data was expressed as withdrawal threshold in grams (g).

### Statistical analysis

All the experiments were repeated three times. Data is presented as mean values ± S.D. Statistical analysis was done by unpaired Student’s t test for two groups and one-way ANOVA for more than two groups using SPSS 13.0 software (SPSS Inc., Chicago, IL, USA). P values < 0.05 were considered significant. The symbols *, **, and *** denote p < 0.05, p < 0.01, and p < 0.001 respectively.

## Results

### Construction and collection of curcumin primed sEV

**Fig. 1:**
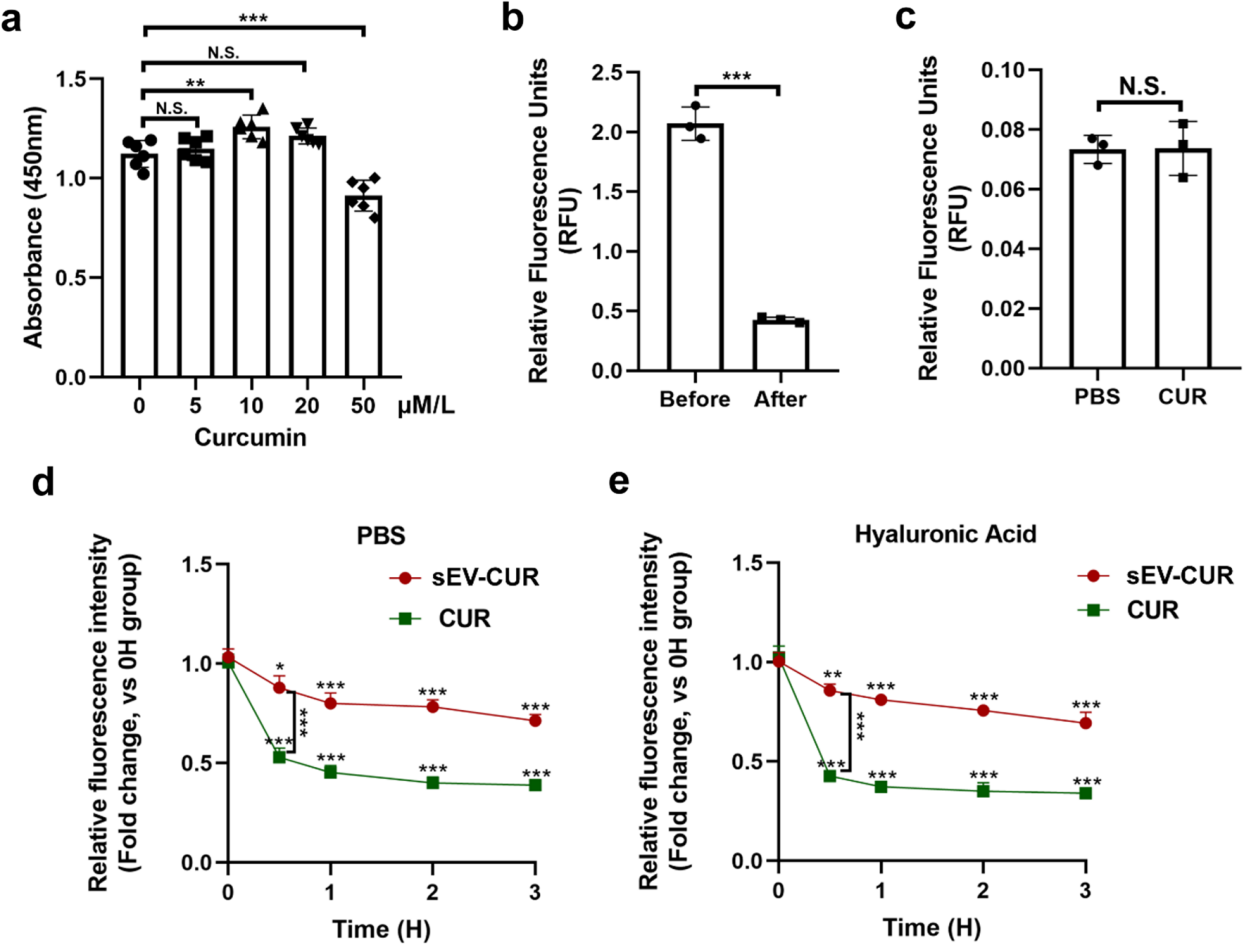
Construction and collection of sEV-CUR. **a** Chondrocyte proliferative ability after treated with different concentrations of curcumin (n = 6); **b** relative fluorescence units of curcumin were detected before and after ultracentrifugation (n = 3); **c** relative fluorescence units were detected after curcumin was isolated from sEV by ultracentrifugation (n = 3); **d** relative fluorescence intensity of sEV and CUR in PBS solution was detected for a 3-h period (n = 3); **e** relative fluorescence intensity of sEV-CUR and CUR in HA solution was detected for a 3-h period (n = 3). N.S., not significant, *P < 0.05, **P < 0.01, ***P < 0.001

To obtain curcumin primed ADMSCs, different concentrations of curcumin were co-incubated with ADMSCs firstly. We found 10 µM/L curcumin significantly promoted ADMSCs proliferation, while other groups showed no significantly protective function (Fig. 1a). Therefore, the optimum concentration of curcumin to treat ADMSCs was 10 µM/L, and it was used in the following experiments. Next, 10 µM/L curcumin was co-incubated with ADMSCs, and curcumin primed sEV were isolated from the culture supernatants by a series of ultracentrifugation. To confirm the curcumin was indeed loaded into ADMSCs-sEV, the fluorescence intensity (Excited: 425 nm; Emitted: 530 nm) of the culture supernatants were detected before ultracentrifugation and the relative fluorescence units were 1.998 ± 0.823 (Fig. 1b). After the isolation, the relative fluorescence units were 0.114 ± 0.053 (Fig. 1b). Therefore, about 82.26% ± 5.25% curcumin was loaded into the sEV. To validate free curcumin was isolated from sEV-CUR by ultracentrifugation, 10 µM/L free curcumin in PBS solution (CUR group) was isolated by ultracentrifugation and the tube bottom was resuspended by PBS. We found the relative fluorescence units of CUR group showed no significant difference to PBS group (Fig. 1c), which verify curcumin was totally isolated from sEV-CUR by ultracentrifugation.

To access the release of free curcumin and sEV-CUR in vitro, the release levels of CUR group and sEV-CUR group were detected in different time points. We found that free curcumin showed faster release rate and obtained a rapid cumulative release of 61% ± 2.9% within 4 h, while the value was up to 84.3% ± 1.7% within 24 h. On the contrary, sEV-CUR controlled the slow release of curcumin and approximately 81.3% ± 3.3% of total curcumin slowly leaked out from sEV into medium within 24 h (Additional file 1: Fig. S1). Moreover, the stability of sEV-CUR was also detected in our study. As we known, curcumin is an agent which highly susceptible to hydrolysis. Therefore, the degradation rates of free curcumin and sEV-CUR were detected in PBS solution and Hyaluronic Acid (HA) solution. We found that free curcumin degraded rapidly than sEV-CUR and the degradation rate of free curcumin exceeded 50% at 1 h in PBS and HA solution, while the degradation rate of sEV-CUR less than 30% at 3 h in PBS and HA solution (Fig. 1d, e). Our result demonstrated that compared with free curcumin, the stability of curcumin was effectively enhanced after being loaded into ADMSCs-sEV.

### Characterization of ADMSCs-sEV and sEV-CUR

**Fig. 2:**
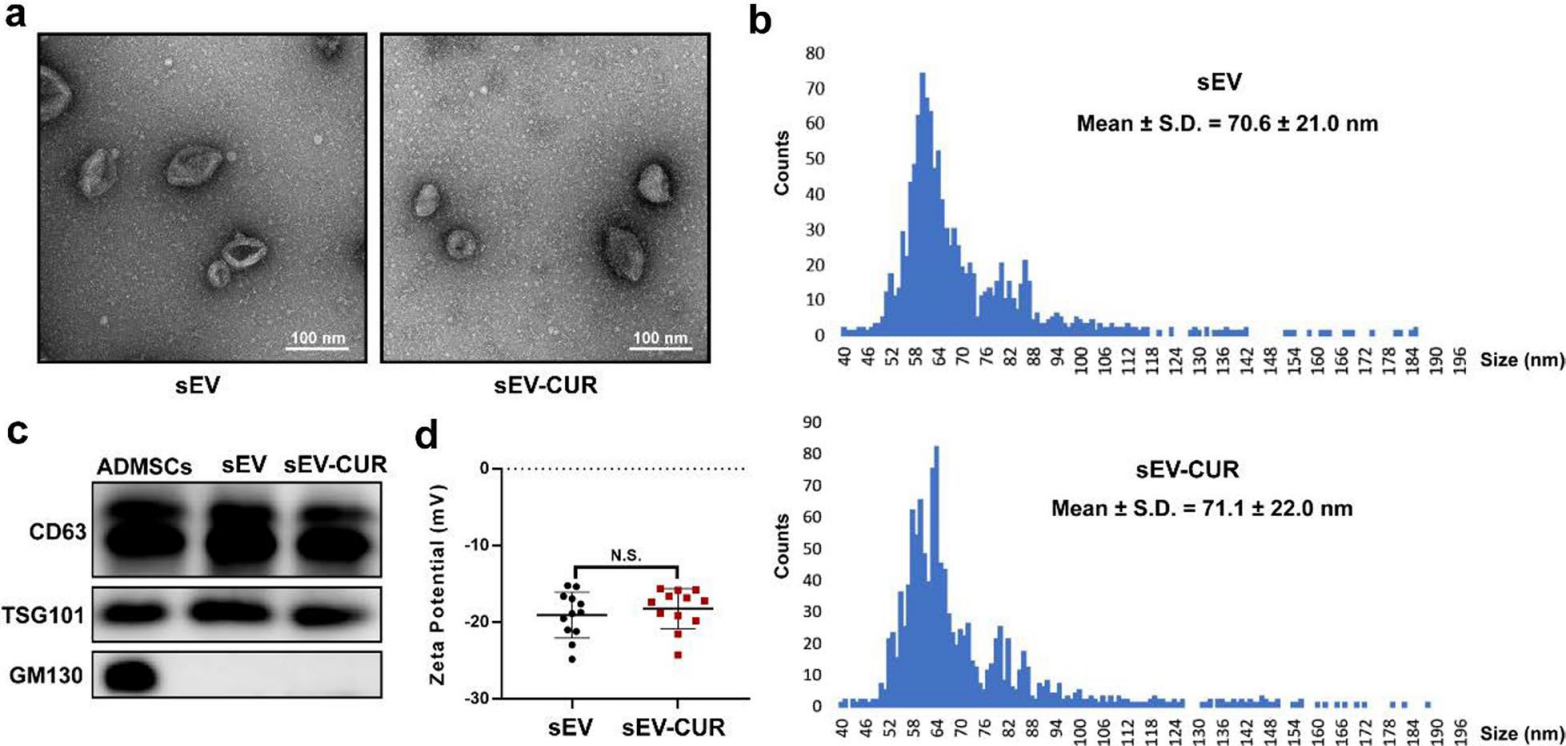
Characterization of sEV-CUR. **a** Representative TEM images of sEV and sEV-CUR, scale bar: 100 nm; **b** average particle diameter distribution of sEV and sEV-CUR was analyzed by a nano-flow cytometry; **c** Western blot analysis of CD63, TSG101, and GM130 from ADMSCs, sEV, and sEV-CUR. CD63 and TSG101: sEV surface marker; GM130: the Golgi marker; **d** Zeta potential of sEV and sEV-CUR (n = 12). N.S., not significant

Then, we evaluated the integrity and morphology of sEV-CUR by a series of experiments. First, our TEM result showed that sEV-CUR exhibited a typical cup-shaped morphology (Fig. 2a). Moreover, the average particle diameter of sEV-CUR group (74.05 ± 2.52 nm) showed no significant difference to sEV group (70.82 ± 3.47 nm) (Fig. 2b and Additional file 1: Fig. S2). The classical markers of sEV, CD63 and TSG101, were detected by western blot analysis to verify that sEV-CUR was isolated from ADMSCs, while GM130 was not detected in sEV group and sEV-CUR group (Fig. 2c). In addition, our result demonstrated that the surface zeta potential of sEV-CUR showed no significantly difference to sEV group (Fig. 2d). Thus, our results suggested that curcumin loaded sEV did not impact the integrity and morphology of ADMSCs-sEV.

### Cellular uptake ability of sEV-CUR

**Fig. 3:**
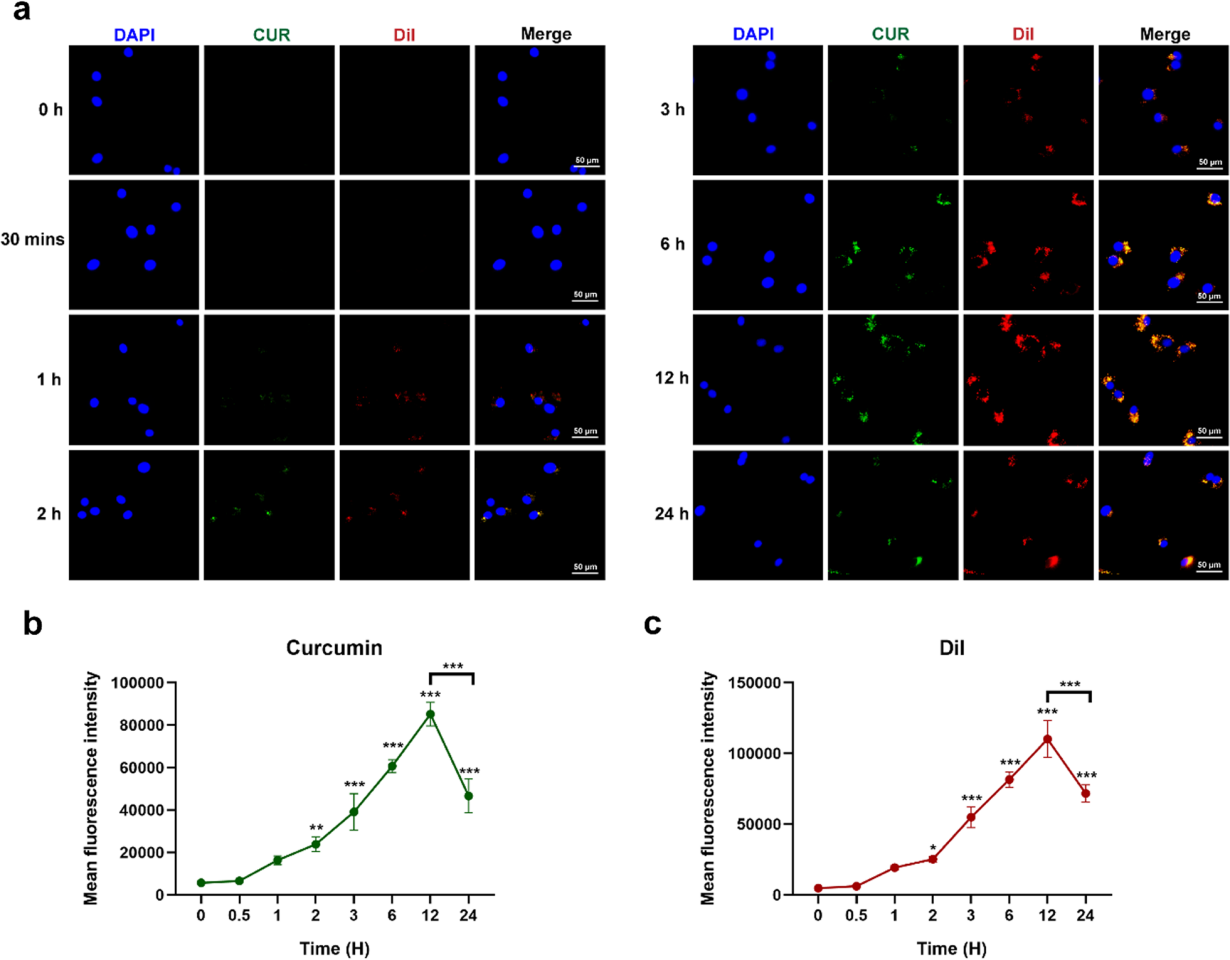
Cellular uptake capacity of sEV-CUR. **a** Fluorescence images of cellular uptake assay was captured by a microscopy during a 24-h period after sEV-CUR treatment; **b** mean fluorescence intensity of curcumin was calculated and analyzed (n = 3); **c** mean fluorescence intensity of DiI was calculated and analyzed (n = 3). *P < 0.05, **P < 0.01, ***P < 0.01

To confirm sEV-CUR can be internalized into chondrocytes, a lipid dye DiI was used to label sEV in our study. First of all, we confirmed that nearly no DiI signal was detected in chondrocytes that were incubated with the PBS resuspensions of 10 µg/mL DiI solution after the same isolation procedures in sEV-CUR group, excluding the possible interference of DiI cluster (Additional file 1: Fig. S3). After co-incubation with DiI labeled sEV-CUR for a 24-h period, chondrocytes were fixed and observed under a confocal microscopy. Our results revealed that the green fluorescence dots of curcumin merged well with the red fluorescence dots of sEV in chondrocytes (Fig. 3a), which means curcumin was loaded into sEV and the sEV-CUR can be taken up by chondrocytes. During the 24-h period, the mean fluorescence intensity of curcumin (Fig. 3b) and DiI (Fig. 3c) were increased along with time and reached the peak at 12 h time point (curcumin: 85205.67 ± 4526.93; DiI: 110,198 ± 10705.15). After 12 h incubation, abundant sEV-CUR were taken up by chondrocytes, while the mean fluorescence intensity was significantly decreased at 24 h time point (curcumin: 46,659 ± 6487.23; DiI: 71725.33 ± 4923.72). Moreover, the fluorescence intensity of sEV and curcumin showed the highly consistent result.

### sEV-CUR enhance the effect of chondrocyte proliferation and extracellular matrix anabolism in vitro

**Fig. 4:**
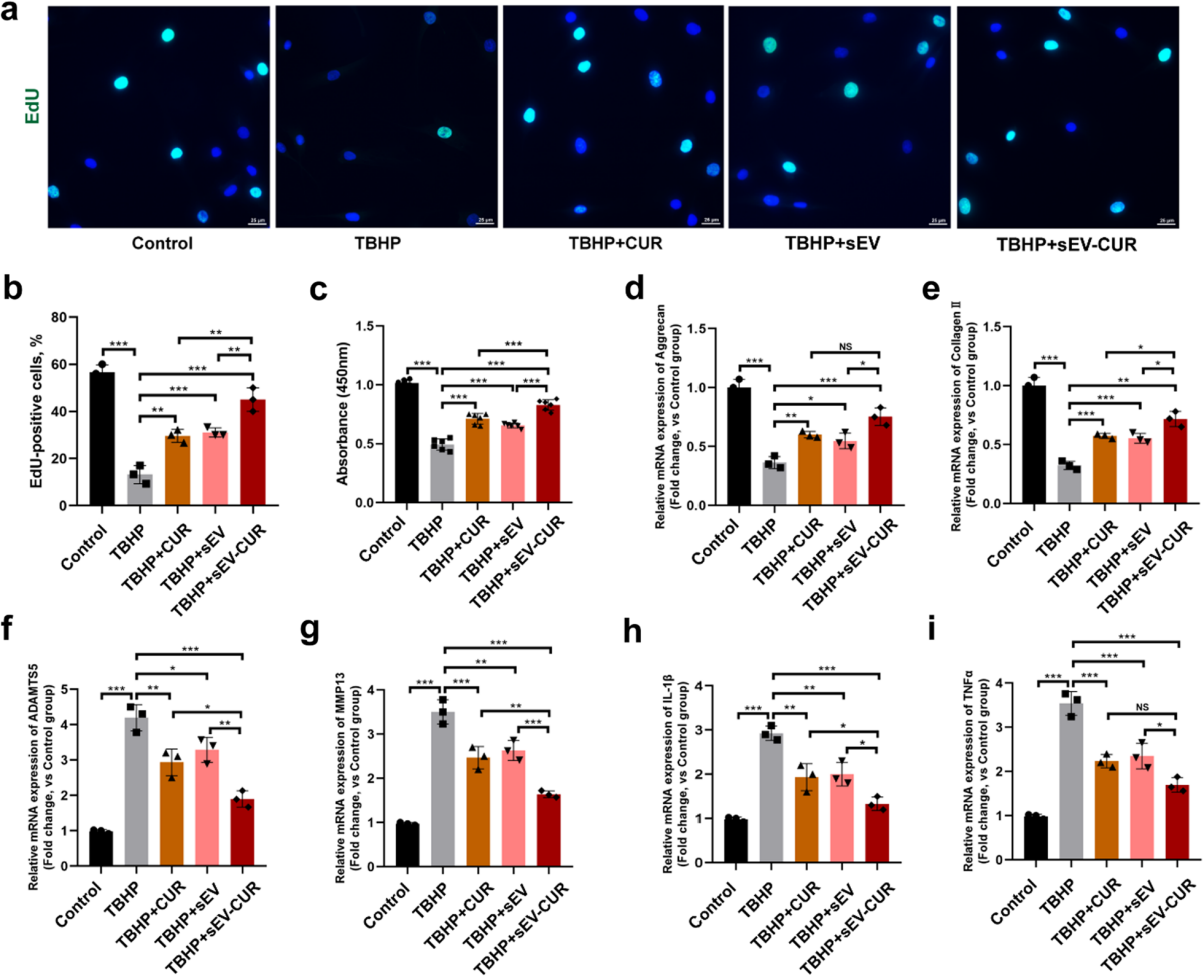
sEV-CUR exhibit enhanced chondro-protective effects in TBHP-induced chondrocytes. **a** Representative EdU staining fluorescence images of curcumin, sEV, or sEV-CUR treated chondrocytes, scale bar: 25 μm; **b** statistical evaluation of fluorescent positive rate after EdU staining (n = 3); **c** the chondrocyte proliferative ability in each group was detected and analyzed by CCK8 assay after 2 days treatment of curcumin, sEV, or sEV-CUR (n = 6); **d**, **e** relative mRNA expression of chondrocyte anabolism related markers aggrecan and collagen II (n = 3); **f**, **g** relative mRNA expression of chondrocyte catabolism related markers ADAMTS5 and MMP13 (n = 3); **h**, **i** relative mRNA expression of inflammatory related markers IL-1β and TNFα (n = 3). NS, not significant, *P < 0.05, **P < 0.01, ***P < 0.001

Next, to evaluate and compare the chondro-protective function of curcumin, sEV, and sEV-CUR, an in vitro chondrocyte OA model was established by using a classical oxidative stress inducer TBHP. The EdU staining assay and CCK8 assay were used to detect the pro-proliferative ability of sEV-CUR in chondrocytes. The EdU-positive cells were markedly decreased after TBHP treatment, while free curcumin, sEV and sEV-CUR all increased the number of EdU-positive chondrocyte after 2 days treatment (Fig. 4a). Specifically, about 30% EdU-positive chondrocytes were observed in curcumin (29.61% ± 2.24%) and sEV (31.1% ± 1.56%) treated group, while sEV-CUR (45% ± 4.08%) showed an enhanced pro-proliferative effect than curcumin and sEV in TBHP-induced chondrocytes (Fig. 4b). The CCK8 assay results were in accordance with the results of EdU staining (Fig. 4c), demonstrating enhanced effects of sEV-CUR on chondrocyte proliferation. Furthermore, we detected the mRNA expressions of chondrocyte extracellular matrix metabolism related markers (aggrecan, collagen II, ADAMTS5, and MMP13) and inflammatory factors (IL-1β and TNFα) after 2 days treatment of curcumin, sEV or sEV-CUR. Our results indicated that sEV-CUR displayed a greater pro-anabolism (aggrecan, collagen II) and anti-catabolism (ADAMTS5, MMP13) function than curcumin and sEV (Fig. 4d–g). Moreover, we also found that curcumin and sEV partially inhibit the expression of IL-1β and TNFα, while sEV-CUR showed a greater anti-inflammatory effect in TBHP-induced chondrocytes (Fig. 4h, i). All these results demonstrated that sEV-CUR maintain chondrocyte metabolism homeostasis more effectively than free curcumin and sEV in vitro.

### sEV-CUR exhibit an enhanced anti-oxidative and anti-apoptosis effect in vitro

**Fig. 5:**
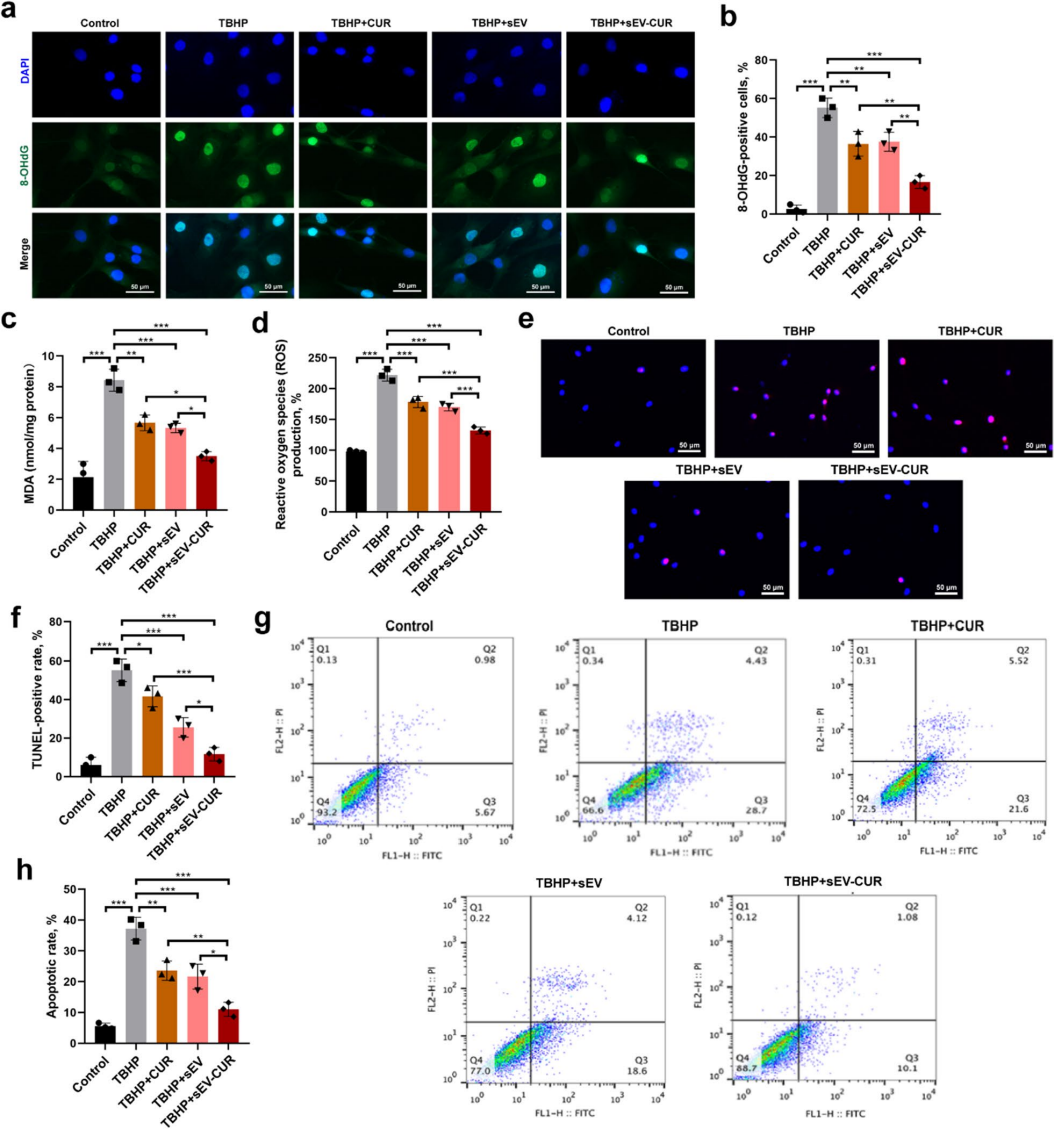
sEV-CUR exert an enhanced anti-oxidative and anti-apoptosis ability. **a**, **b** Immunofluorescence staining and statistical analysis of oxidative stress related marker 8-OHdG in each group (n = 3), scale bar: 50 μm; **c** MDA level was evaluated in each group (n = 3); **d** ROS level in each group was detected after curcumin, sEV, or sEV-CUR treatment for 2 days (n = 3); **e**, **f** representative images and quantitative analysis of TUNEL staining analysis to detect chondrocyte apoptosis (Red) different groups, scale bar, 50 μm; **g**, **h** chondrocyte apoptotic degree in each group was detected and analyzed by flow cytometry assay (n = 3). *P < 0.05, **P < 0.01, ***P < 0.001

Given the essential role of oxidative stress on mediating chondrocyte apoptosis in OA initiation and progression, the level of oxidative stress in each group was detected by immunofluorescence microscopy, ROS and MDA detection. First, a classical oxidative stress marker 8-OHdG was detected and our result revealed that sEV-CUR effectively enhanced the anti-oxidative effect of curcumin and sEV in TBHP-induced chondrocytes, as 8-OHdG positive cells were significantly decreased after sEV-CUR treatment (Fig. 5a, b). Next, we detected the levels of ROS and MDA in different groups and we found the levels of ROS and MDA were markedly down-regulated in sEV-CUR group (Fig. 5c, d), which indicated that the anti-oxidative effect significantly improved after curcumin was loaded into sEV. Then, the anti-apoptotic effects of free curcumin, sEV, and sEV-CUR on TBHP treated chondrocytes were analyzed by Annexin V-FITC/PI flow cytometry, TUNEL staining, and immunofluorescence staining of cleaved caspase3. The apoptotic rate was partially decreased after curcumin (23.58% ± 2.55%) and sEV (21.67% ± 3.32%) treatment, while sEV-CUR (11.02% ± 1.87%) inhibited the chondrocyte apoptosis more effectively than curcumin and sEV in vitro (Fig. 5e, f). In addition, TUNEL staining showed similar results with the results of flow cytometry. We found the TUNEL-positive cells were significantly down-regulated after sEV-CUR treatment (Fig. 5g, h). Immunofluorescence staining of cleaved caspase3 also showed that sEV-CUR (15.62% ± 2.00%) decrease the cleaved caspase3-positive cells more efficiently than curcumin (33.44% ± 4.04%) and sEV (29.69% ± 3.47%) in TBHP-induced chondrocytes (Additional file 1: Fig. S4). Thus, our results indicated the enhanced chondro-protective effects of sEV-CUR are closely related with the anti-oxidative and anti-apoptosis effects in vitro.

### sEV-CUR enhance the cartilage protective effects in ACLT-induced OA mice

**Fig. 6:**
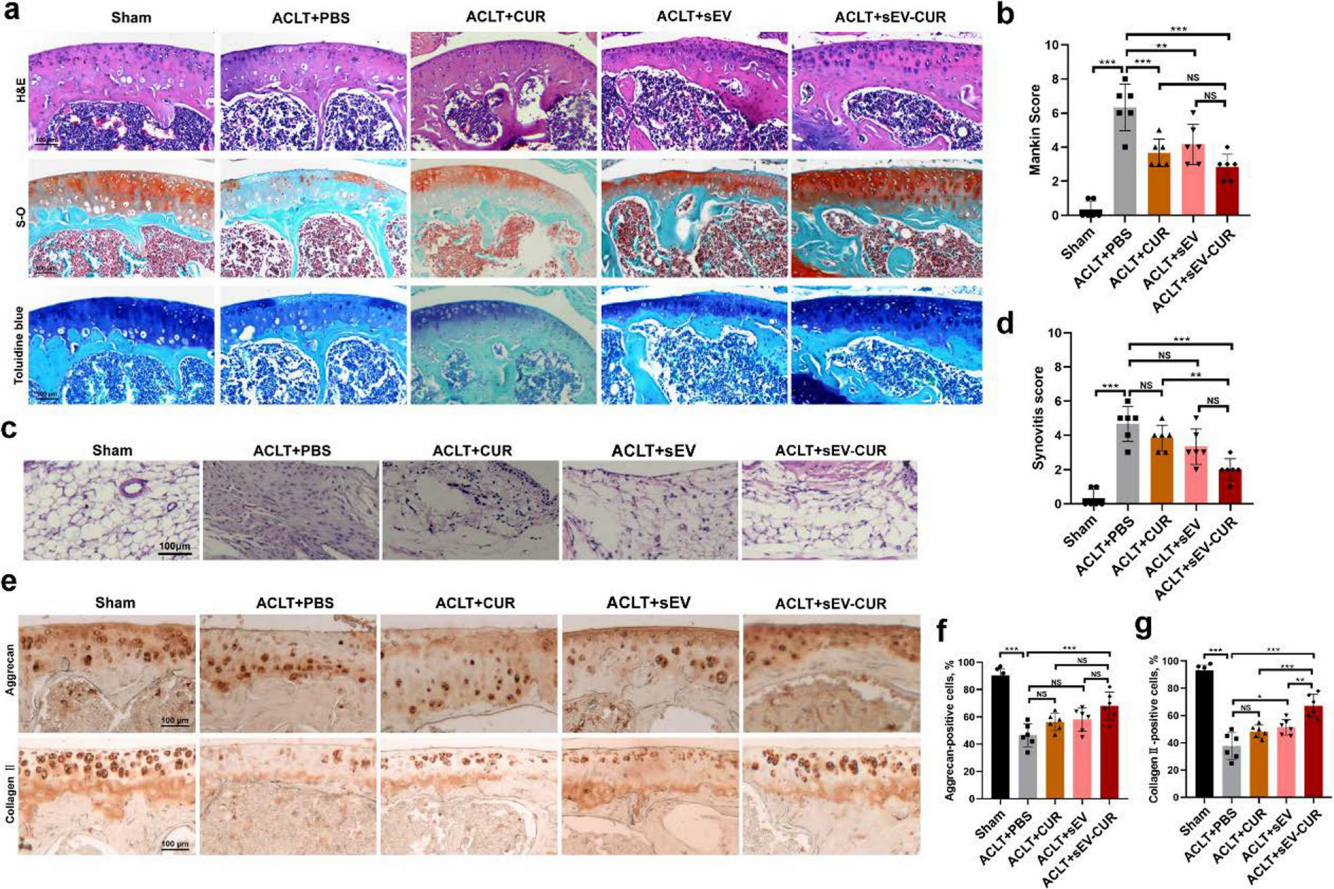
sEV-CUR exhibit an enhanced protective function in ACLT-induced OA mice. **a** Representative images of H&E, Safranin O-Fast Green (S–O) staining, and Toluidine blue staining of articular cartilage in each group, scale bar: 100 μm; **b** statistic data of Mankin scores in each group (n = 6); **c** representative image of H&E staining of synovium in each group, scale bar: 100 μm; **d** statistical analysis of synovitis score in each group (n = 6); **e** representative image of immunohistochemistry staining for extracellular matrix anabolism biomarker aggrecan and collagen II in each group, scale bar: 100 μm; **f**, **g** quantitative analysis of immunohistochemical staining of aggrecan and collagen II (n = 6). NS, not significant, *P < 0.05, **P < 0.01, ***P < 0.001

Next, we further investigated whether the improved function of sEV-CUR was available in ACLT-induced OA mice model. As we known, frequent and long-term intra-articular injections bring about many problems, such as soft tissues injuries and joint infection. Thus, reducing the injection frequency is essential for the clinical application of MSCs-sEV for OA patients. Therefore, we explored the cartilage protective effects of sEV-CUR in vivo after biweekly intra-articular injection for 4 weeks. After treatment, H&E staining, S–O staining, and toluidine blue staining were performed to evaluate the cartilage degeneration and synovial inflammation in each group. We found obvious cartilage destruction and loss in the ACLT group, and those features were partially ameliorated in free curcumin and sEV treated group. However, sEV-CUR more efficiently alleviated cartilage degeneration and abrasion in OA mice (Fig. 6a). Histological analysis was applied to quantify the severity of OA in a blinded manner according to the Mankin scoring system. Curcumin (3.67 ± 0.75) and sEV (4.17 ± 1.07) partially decrease the Mankin score of ACLT mice (6.33 ± 1.25), while the Mankin score in sEV-CUR group is 2.83 ± 0.69 (Fig. 6b). Our results also demonstrated that sEV-CUR (2 ± 0.58) alleviated synovial inflammation in ACLT-induced OA joint more efficiently than free curcumin (3.83 ± 0.69), but showed no statistical difference with sEV group (Fig. 6c, d). Then, IHC staining for anabolism-related protein (aggrecan and collagen II) was carried out to verify whether sEV-CUR exert an enhanced cartilage protective effect in ACLT-induced mice. Our IHC staining results showed that the expression of aggrecan was significantly increased in ACLT-induced mice (46.5% ± 7.68%) after the treatment of sEV-CUR (68% ± 9.26%), while CUR group (56.17% ± 5.76%) and sEV group (58% ± 7.85%) showed no significant difference to ACLT group (Fig. 6e, f). Moreover, IHC staining for collagen II also indicated that sEV-CUR exert an improved chondro-protective than free curcumin and sEV (Fig. 6g).

### sEV-CUR effectively alleviate oxidative stress, chondrocyte apoptosis, and OA-related pain in mice

**Fig. 7:**
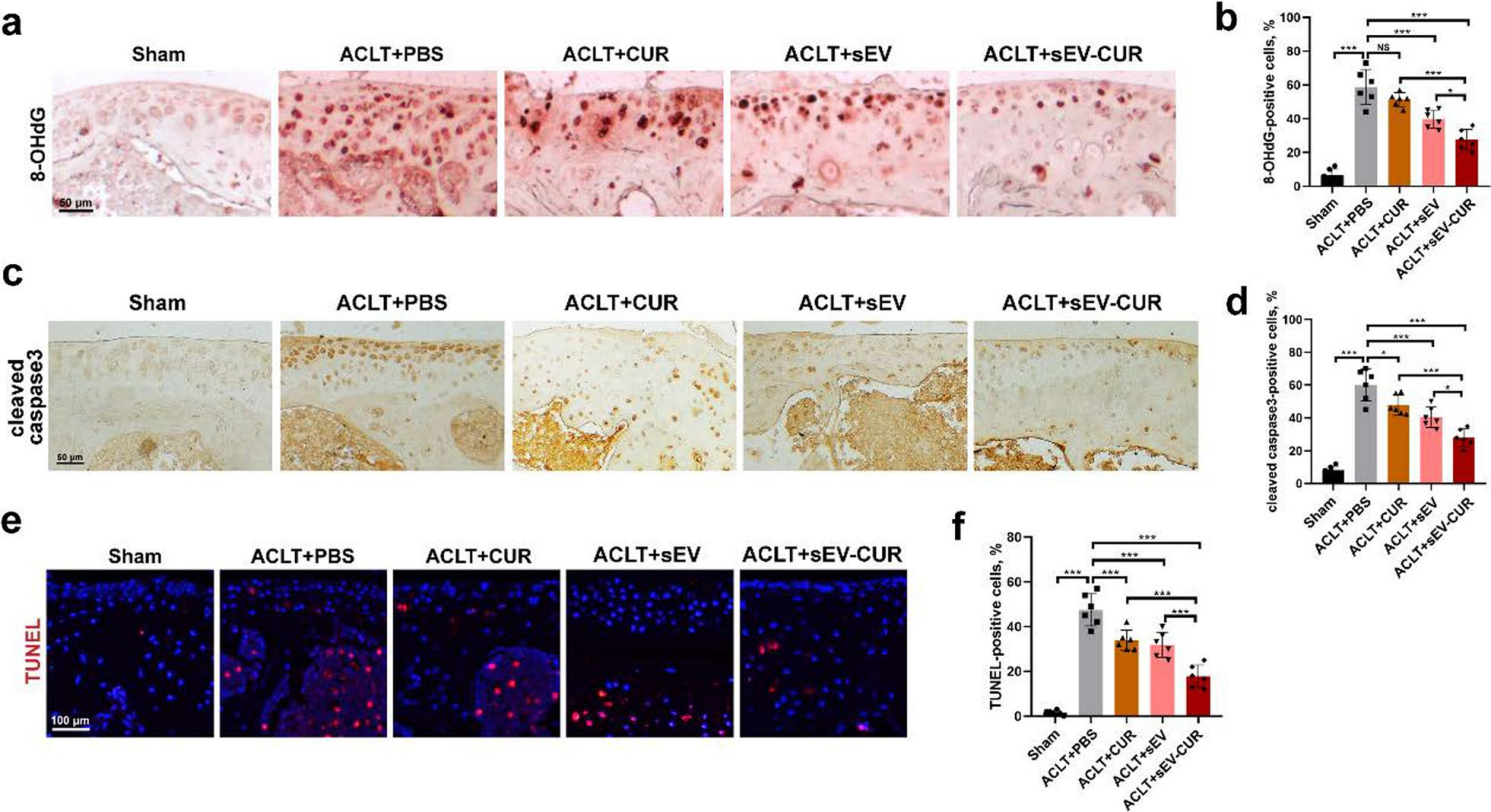
sEV-CUR exert an improved anti-oxidative and anti-apoptosis ability in vivo. **a**, **b** Representative images and quantitative analysis of immunohistochemistry staining for a classical oxidative stress biomarker 8-OHdG in each group (n = 6), scale bar: 50 μm; **c**, **d** representative images and quantitative analysis of immunohistochemistry staining for a classical apoptosis biomarker cleaved caspase3 in each group (n = 6), scale bar: 50 μm; **e**, **f** representative images and quantitative analysis of TUNEL staining analysis to detect chondrocyte apoptosis (Red) in vivo in each group (n = 6), scale bar, 100 μm. NS, not significant, *P < 0.05, ***P < 0.001

To further validate whether the chondro-protective of sEV-CUR is associated with the anti-oxidative and anti-apoptosis effect in vivo, oxidative stress and apoptosis related markers were detected by IHC staining. First, oxidative stress related marker 8-OHdG was detected by IHC staining. Our results indicated that the expression of 8-OHdG was markedly up-regulated in articular cartilage of ACLT-induced mice (58.83% ± 9.41%), while sEV (39.83% ± 4.88%) and sEV-CUR (27.5% ± 5.56%) both decreased the expression of 8-OHdG (Fig. 7a, b). However, sEV-CUR showed an enhanced anti-oxidative effect than free curcumin and sEV, as the expression of 8-OHdG in sEV-CUR group was lower than that in CUR group and sEV group. Furthermore, the anti-apoptosis effects of sEV-CUR and sEV in articular cartilage of ACLT-induced mice were also detected by IHC staining. Free curcumin (47.83% ± 5.58%) and sEV (40.45% ± 5.59%) both decreased the expression of apoptotic-related marker cleaved caspase3 in ACLT-induced mice (59.98% ± 8.91%), while sEV-CUR (28% ± 4.97%) showed a better anti-apoptosis effect than free curcumin and sEV (Fig. 7c, d, Additional file 1: Fig. S5). TUNEL staining showed similar results with cleaved caspase3 IHC staining. We found that TUNEL-positive chondrocytes in ACLT-induced mice cartilage (47.5% ± 6.6%) were significantly down-regulated after sEV-CUR treatment (17.78% ± 4.61%) (Fig. 7e, f).

As pain is the main symptom of OA patients, so we also evaluated pain related mechanical sensitivity after the treatment of curcumin, sEV or sEV-CUR in ACLT-induced mice. After 2 weeks treatment, all three groups did not effectively enhance the withdrawal threshold of ACLT mice (Additional file 1: Fig. S6). After 4 weeks treatment, we found sEV-CUR (7.5 ± 1.89) effectively increased the withdrawal threshold of ACLT-induced mice (4 ± 1.63), while the withdrawal threshold of curcumin (4.67 ± 1.25) and sEV (5.33 ± 1.6) still showed no statistical difference with it in ACLT + PBS group (Fig. 7f).

## Discussion

MSCs-sEV have emerged as a promising candidate for therapeutic application in degenerative diseases by promoting tissue repair and regeneration. Recent studies demonstrated various MSCs-sEV have showed protective function on chondrocytes and articular cartilage in vitro and in vivo [13], indicating a great therapeutic potential in OA treatments. However, owing to the dense cartilage matrix and the fast clearance system in the joint cavity [40–42], MSCs-sEV were intra-articularly injected at least once a week in these studies. Moreover, in these studies, sEV are intra-articularly injected in the early stage of OA, while articular cartilage is relatively intact in this stage, causing more injected dose and frequent injection number. Therefore, we need to modify and optimize MSCs-sEV to improve their bioavailability and therapeutic function on OA cartilage. In our study, we constructed curcumin primed sEV, and we found that sEV-CUR effectively promoted proliferative ability and anabolism activity in TBHP-induced chondrocytes. In addition, we first provide evidence that sEV-CUR alleviated oxidative stress and apoptosis more efficiently than free curcumin and ADMSCs-sEV. In ACLT-induced OA mice, our results first demonstrated that sEV-CUR exerted an enhanced cartilage protective function than free curcumin and sEV, as sEV-CUR successfully alleviated cartilage loss and degeneration in OA mice. Moreover, we also found that sEV-CUR showed a better anti-oxidative and anti-apoptosis ability than free curcumin and sEV in OA mice. Thus, these above results revealed that sEV-CUR exhibited an enhanced chondro-protective effects in vitro and in vivo. Collectively, these results suggested that sEV-CUR have potential as a promising therapeutic approach against OA.

As we known, curcumin is classical anti-oxidative and anti-inflammatory agent which was widely used in the treatment of many inflammatory and degenerative diseases [43, 44]. Previous studies showed that curcumin exerts chondro-protective effects by regulating oxidative stress [45], endoplasmic reticulum stress [30], inflammation [46], autophagy [47] in different OA models. However, the administration of curcumin was relatively high-dose and frequent, it may cause intra-articular infection, soft tissue injuries, side-effects of other organs, and may other problems. A recent study demonstrated that curcumin pre-treated MSCs-sEV inhibited chondrocyte apoptosis and slowed OA progression [34]. However, in this study, whether curcumin was loaded into the sEV and the loading efficiency of free curcumin in sEV was unclear, need to be further clarified. In our study, we found sEV-CUR showed a more stable property in PBS or HA solution than curcumin. We also found about 82.26% ± 5.25% free curcumin was loaded into the ADMSCs-sEV. Moreover, our results indicated the green fluorescence of curcumin merged well with DiI labeled sEV membrane, indicating that curcumin was loaded into the sEV-CUR. It provides theoretical basis for possible clinical translation of sEV-CUR in the future.

Oxidative stress is an essential reason for the initiation and progression of OA. In previous articles about the chondro-protective effects of curcumin pre-treated sEV in OA treatment [34, 48], whether the anti-oxidative function was associated with protective effects is unclear. In our study, we found that sEV-CUR exerted an enhanced anti-oxidative ability than free curcumin and ADMSCs-sEV, as sEV-CUR significantly alleviated oxidative stress and cell apoptosis in TBHP-induced chondrocytes. Moreover, the levels of ROS and MDA were also decreased after sEV-CUR treatment. However, the molecular mechanism of sEV-CUR on the anti-oxidative effect needs to be further investigated. In addition, more studies need to be conducted to uncover the role of specific sEV contents (including miRNAs, mRNA, lncRNA, DNA, proteins, and lipid) in regulating oxidative stress and chondrocyte apoptosis. Moreover, there are some other limitations should be acknowledged in our study. A long-term pharmacological study and relevant validation experiments in large animals should be conducted before sEV-CUR are translated into the clinic. Moreover, since it is still not feasible to perform biweekly intra-articular injection of sEV-CUR in OA patients, further surface modification of sEV-CUR targeting articular cartilage should be addressed in the future research.

**Fig. 8:**
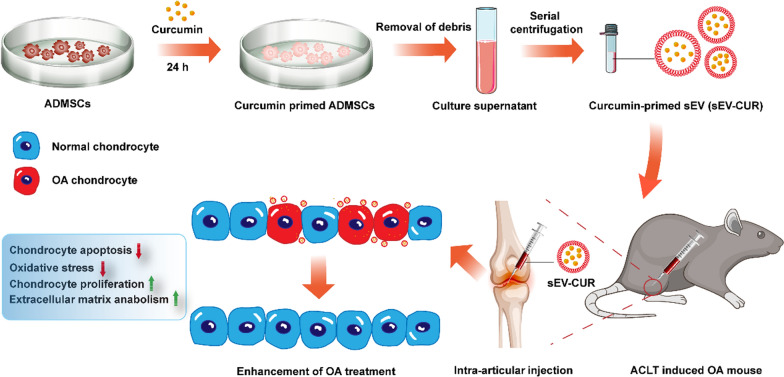
The therapeutic effects of sEV-CUR in ACLT-induced OA mouse

In conclusion, our study indicated the feasibility of sEV-CUR to promote chondrocyte proliferation and alleviate cartilage destruction in ACLT-induced OA mice (Fig. 8). We provide evidence that sEV-CUR exerted enhanced chondro-protective effects by down-regulating oxidative stress and chondrocyte apoptosis. In addition, our in vivo studies showed that sEV-CUR showed significantly enhanced cartilage therapeutic efficacy in ACLT-induced OA mice with reduced injection frequency. Collectively, these results suggested that sEV-CUR have potential as a promising therapeutic approach against OA.

## Supplementary Information

**Additional file 1: Figure S1.** In vitro releaseprofile of free curcumin and sEV-CUR in PBS for 24h. **Figure S2.** Averageparticle diameter of sEV and sEV-CUR. **FigureS3.** Detection of thefluorescent signal of DiI clusters in chondrocytes. **Figure S4.** Immunofluorescencestaining of cleaved caspase3 in vitro.**Figure S5.** Immunohistochemistry staining of cleaved caspase3in vivo. **Figure S6.** The effectof sEV-CUR on mechanical sensitivity in ACLT-induced mice.

## Data Availability

The data that support the findings of this study are available from the corresponding authors upon reasonable request.
