# Supplementary material for: Curcumin primed ADMSCs derived small extracellular vesicle exert enhanced protective effects on osteoarthritis by inhibiting oxidative stress and chondrocyte apoptosis
Source: J Nanobiotechnology. 2022 Mar 9;20:123. doi: 10.1186/s12951-022-01339-3 (PMC8905866; doi:10.1186/s12951-022-01339-3)
Supplement: Supplementary file 1 — Additional file 1: Figure S1. In vitro releaseprofile of free curcumin and sEV-CUR in PBS for 24h. Figure S2. Averageparticle diameter of sEV and sEV-CUR. FigureS3. Detection of thefluorescent signal of DiI clusters in chondrocytes. Figure S4. Immunofluorescencestaining of cleaved caspase3 in vitro.Figure S5. Immunohistochemistry staining of cleaved caspase3in vivo. Figure S6. The effectof sEV-CUR on mechanical sensitivity in ACLT-induced mice. [file 12951_2022_1339_MOESM1_ESM.docx]

**Additional file 1 for**

**Curcumin primed ADMSCs derived small extracellular vesicle exert enhanced protective effects on osteoarthritis by inhibiting oxidative stress and chondrocyte apoptosis**

Chen Xu^1,*^, Zanjing Zhai^1,*^, Hua Ying^1^, Lin Lu^2^, Jun Zhang^1,#^, Yiming Zeng^1,#^

^1^ Shanghai Key Laboratory of Orthopedic Implants, Department of Orthopedics, Ninth People’s Hospital, Shanghai Jiao Tong University School of Medicine, Shanghai 200011, China.

^2^ Department of Plastic and Reconstructive Surgery, Shanghai Ninth People's Hospital, Shanghai Jiao Tong University School of Medicine, Shanghai 200011, China.

*Chen Xu and Zanjing Zhai contributed equally to this work.

#Correspondence authors:

Yiming Zeng, xyhz29@163.com;

Jun Zhang, jeferry717717@163.com.

**
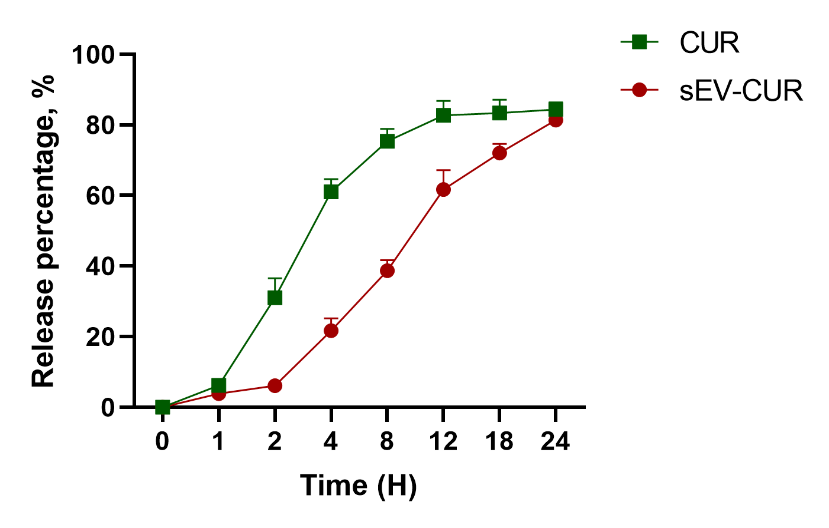
**

**Figure S1. In vitro release profile of free curcumin and sEV-CUR in PBS for 24 hours.**


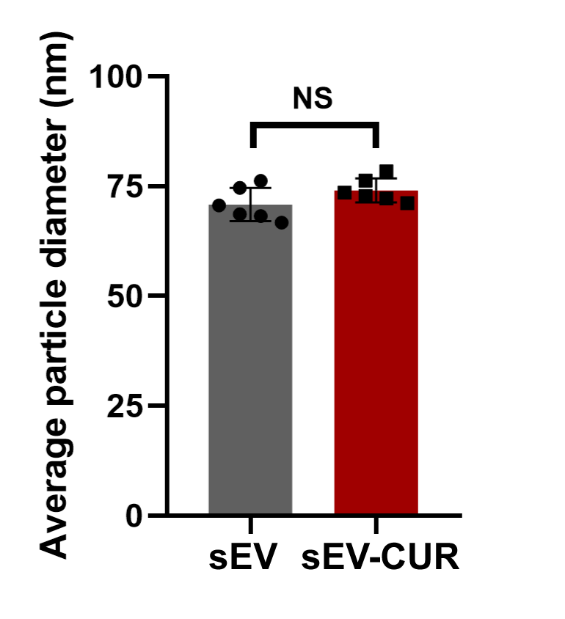


**Figure S2. Average particle diameter of sEV and sEV-CUR.**

**
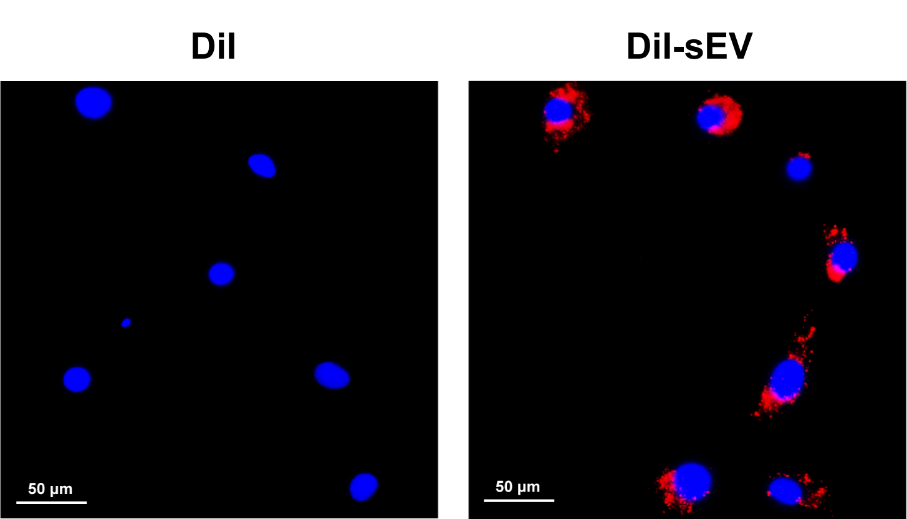
**

**Figure S3. Detection of the fluorescent signal of DiI clusters in chondrocytes.**


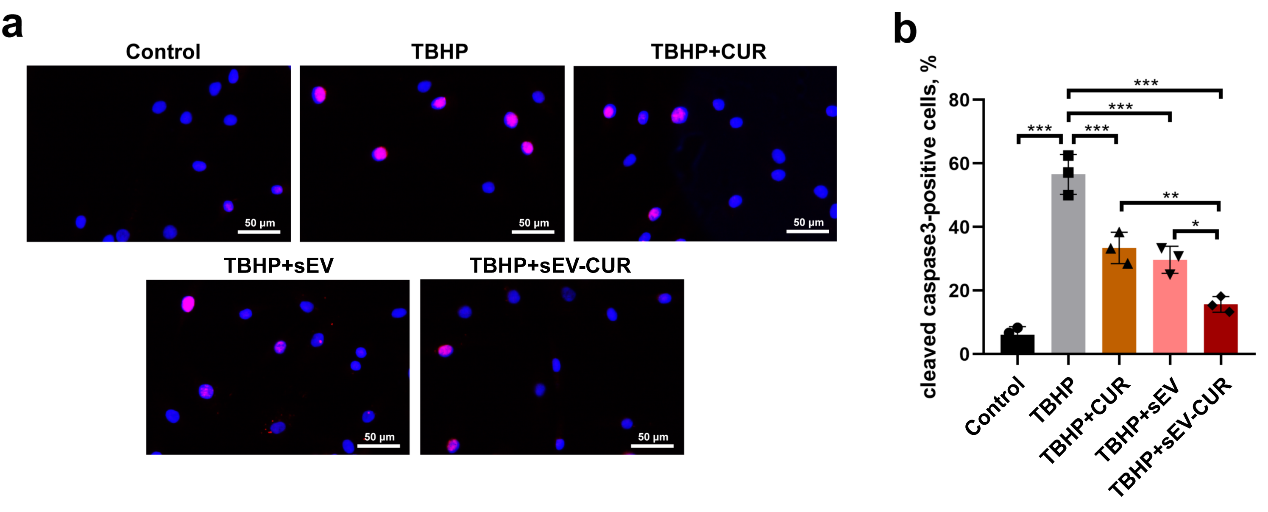


**Figure S4. Immunofluorescence staining of cleaved caspase3 *in vitro*.**

(a) Representative immunofluorescence images of cleaved caspase3 in each group, scale bar: 50 μm; (b) Statistical evaluation of fluorescent positive after EdU staining (n = 3). *P < 0.05, **P < 0.01, ***P < 0.001.


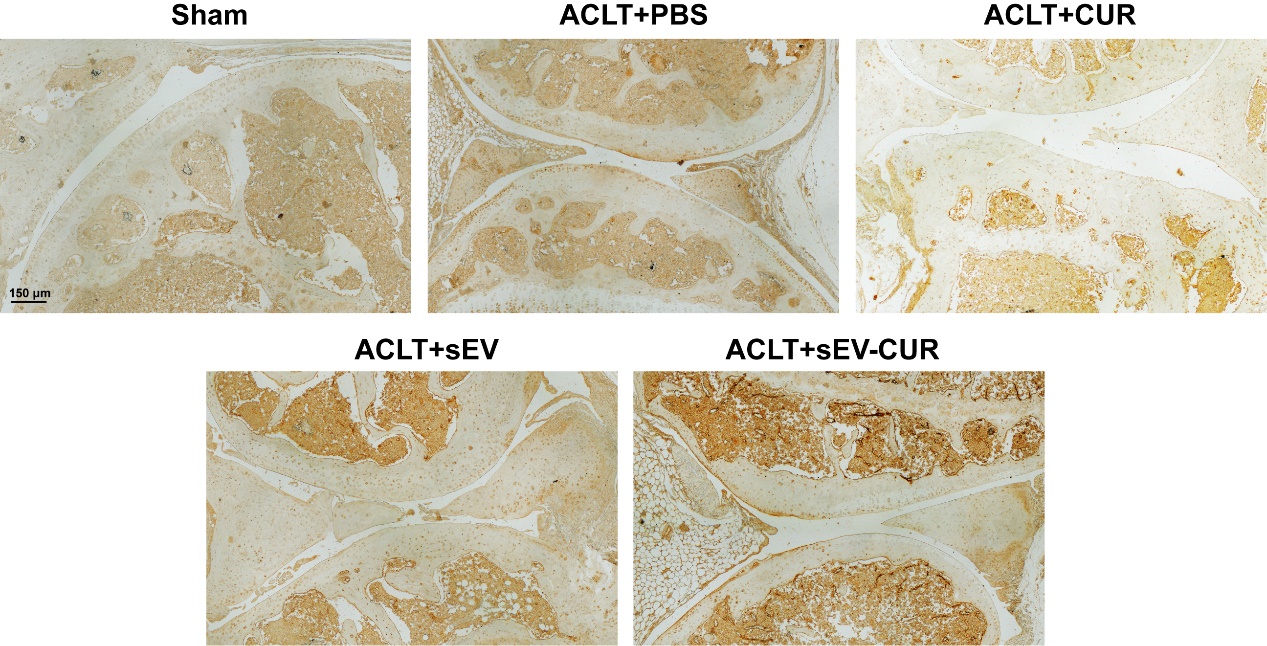


**Figure S5. Immunohistochemistry staining of cleaved caspase3 *in vivo*.**


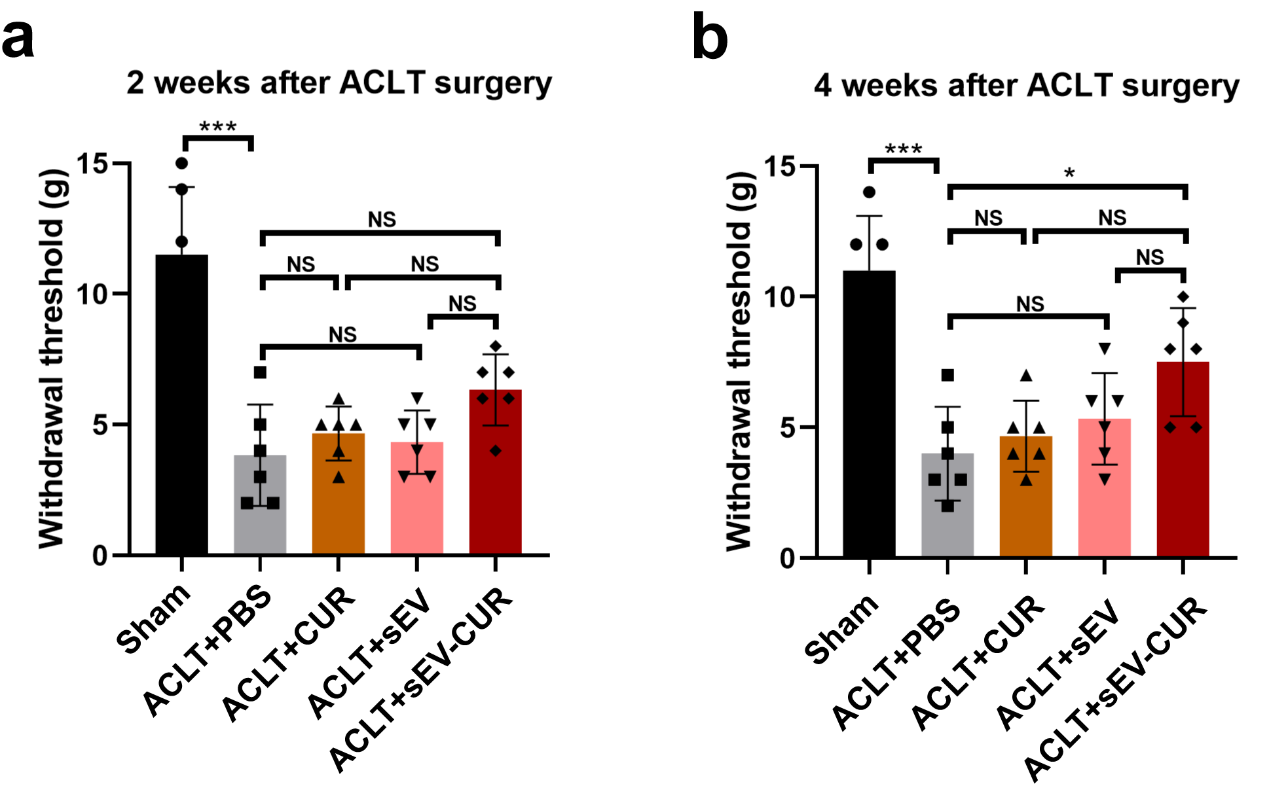


**Figure S6. The effect of sEV-CUR on mechanical sensitivity in ACLT-induced mice.**

Mechanical sensitivity in each group was measured and analyzed at 2 weeks (a) and 4 weeks (b) after ACLT surgery (n = 6). WT, withdrawal threshold. NS, not significant, *P < 0.05, ***P < 0.001.
